# Acute traumatic unilateral cervical C4–C5 facet dislocation in pediatric toddlers

**DOI:** 10.1186/s12891-019-3019-9

**Published:** 2020-01-03

**Authors:** Wongthawat Liawrungrueang, Rattanaporn Chamnan, Weera Chaiyamongkol, Piyawat Bintachitt

**Affiliations:** Department of Orthopedics, Faculty of Medicine, Prince of Songkla University, 15 Karnjanavanich Road, Songkhla, Hat Yai, 90112 Thailand

**Keywords:** Acute unilateral facet dislocation, Perched facet, Pediatric

## Abstract

**Background:**

The present study is to highlight the challenges in managing cervical spine injuries in toddlers (less than 4 years of age) without neurological deficit. Cases of unilateral cervical C4–C5 facet dislocation in toddlers are very rare.

**Case presentation:**

A 3-year-old girl suffered cervical spine injury after a motor vehicle collision with unilateral C4–C5 facet dislocation without neurological deficit. Magnetic resonance imaging (MRI) showed no spinal cord injury, Frankel grade E. Initial management was cervical spine protection. Definite treatment and complication were discussed with the patient’s parents before closed reduction maneuver with minerva cast was applied under sedation. The patient showed no complication after closed reduction and the cervical spine had aligned well in radiographs. The minerva cast was removed at 8 weeks, at which point neck muscle stretching rehabilitation program started. At one-year follow up, the child was asymptomatic, had full active cervical motion and good function. In radiographs, the cervical spine had normal alignment and was healed.

**Conclusions:**

Unilateral cervical facet dislocation in toddlers is very rare. Closed reduction maneuver and the minerva cast applied were optional in this case. The parents were highly satisfied with the effective treatment and outcome.

## Background

Cervical spinal cord injury without radiographic abnormalities (SCIWORA) is more commonly seen in the pediatric age group than in adults. Incidences have been reported as 13–19% of spinal injuries in children [1–4]. However, the trauma of C3-C7 lower cervical area is especially seen in adolescence and the advanced childhood period. While lower cervical area trauma ratios are 20–30% in children under 9 years of age, this rises to 70–75% in adolescence and advanced childhood. In the study of McGrory et al. [5] Facet dislocations can be unilateral or bilateral. They generally develop in connection to the hyperflexion traumas accompanied by rotation. Patients show symptoms with reticular and/or spinal cord base findings. Compared with unilateral, bilateral facet dislocations are more unstable pathologies [6]. Even though diagnosis can be made by lateral radiography, computed tomography scan (CT) and magnetic resonance imaging (MRI) are necessary to finalize the diagnosis. In early diagnosis cases, reduction can be ensured by traction. In cases in which reduction is ensured, 2–4 months immobilization must be provided by halo or minerva orthosis. In cases where reduction cannot be ensured, reduction and fusion indication appears with anterior or posterior surgeries [6, 7]. Previously reviewed literature and reports showed unilateral cervical C4–C5 facet dislocation in toddlers is very rare and there are even less reports concerning the treatment process. The purpose of this study was to highlight the challenges in managing cervical spine injuries in toddlers (less than 4 years of age) without neurological deficit. Cases of unilateral cervical C4–C5 facet dislocation in toddlers is very rare. The authors describe the management of an acute pediatric unilateral facet dislocation.

## Case presentation

**Fig. 1:**
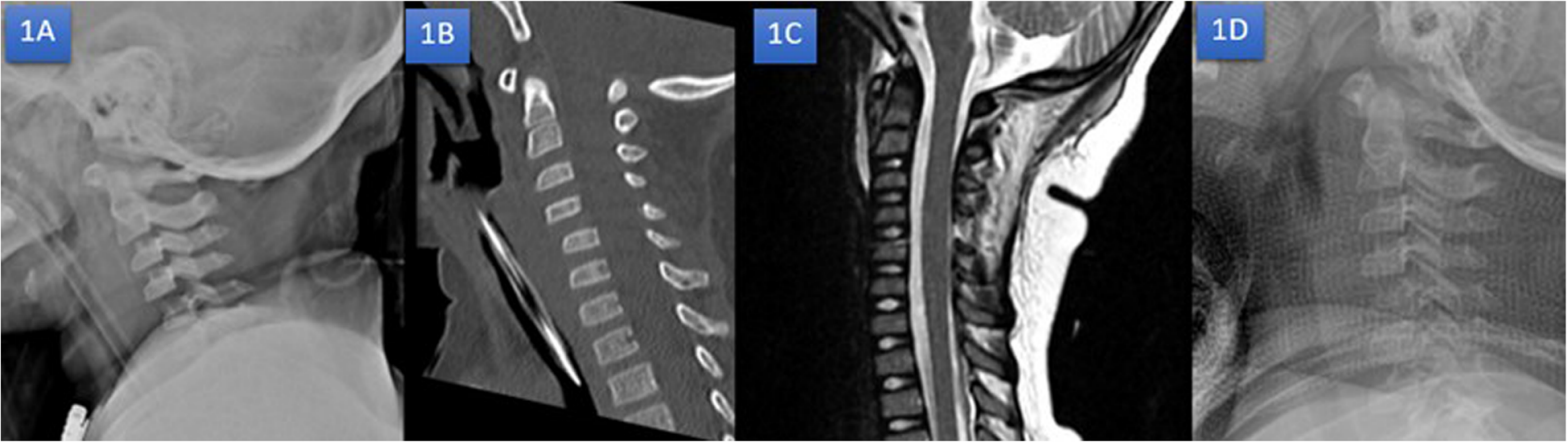
Radiographs images; Lateral radiograph x-ray (1A), sagittal radiograph of CT (1B) and sagittal radiograph of T2 weighted MRI (1C) showed antrolesthesis of C4 on C5 with 25% translation without spinal cord injury. Repeated lateral radiograph x-ray after closed reduction maneuver with minerva casting (1D)

**Fig. 2:**
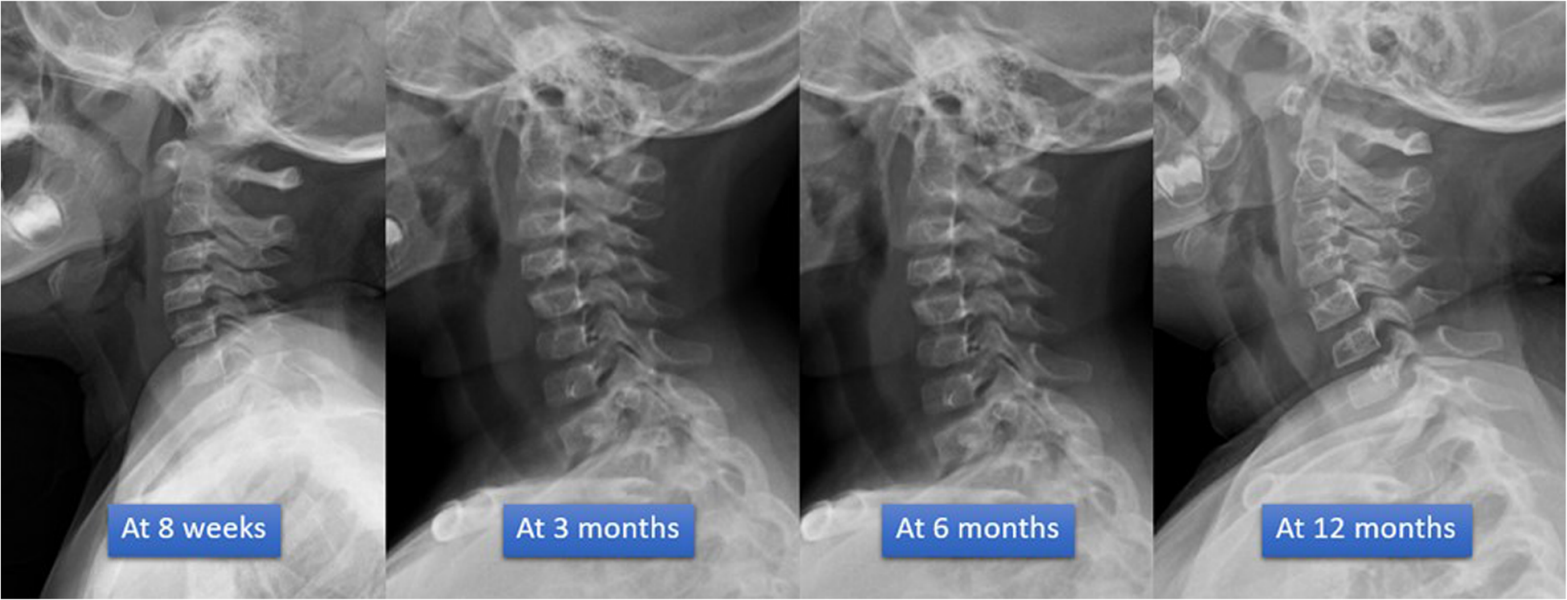
Radiographs images after removing minerva cast; showing the cervical spine had improved alignment and healed in follow up radiographs

A 3-year-old girl suffered cervical spine injury after a motor vehicle collision while sitting in the car without wearing a seatbelt. ATLS protocol was performed on the patient at a local hospital and she was referred to the emergency department at author’s hospital within 12 h of the injury. The pediatric surgeon and our orthopedic team re-evaluated that the status of the patient showed head injury with alteration of consciousness, intubation, cervical spine protection with hard collar and first rib fracture without pneumohemothorax. The emergency radiographs x-ray and CT brain including cervical spine showed no intracerebral hemorrhage but the cervical spine suffered unilateral cervical C4–C5 facet dislocation. Radiographic features showed anterior dislocation of the affected vertebral body less than the vertebral body in anterior posterior diameter, discordant rotation above and below involved level, facet within intervertebral foramen on oblique view, widening of the disk space and “Bat wing sign” appearance of the overriding facet (Fig. 1a-c). The patient was taken to the pediatric intensive care unit (PICU) for resuscitation and closed monitoring after hemodynamic was stable. The patient was evaluated by MRI for preoperative planning, and no spinal cord injury was visible. In the next 24 hours, the patient’s neurovascular status examination was fully conscious and no neurological deficit (Frankel grade E) then extubation followed. The team discussed with her parents treatment plan and complication. Her parents then denied surgery. The authors applied closed reduction maneuver with minerva cast under sedation. The patient was in Frankel grade E without complication after closed reduction and the cervical spine had good alignment in radiographs (Fig. 1d). The minerva cast was removed at 8 weeks, at which point neck muscle stretching rehabilitation program started. At one-year follow up, the child was asymptomatic, had full active cervical motion and good function. The cervical spine showed normal alignment and had healed in follow up radiographs (Fig. 2). Her parents were highly satisfied with our effective treatment and outcome.

## Discussion and conclusions

The study subjects is a traumatic pediatric unilateral cervical facet dislocation in toddler (18 months – 3 yrs.) which is a very rare condition. The most common causes of pediatric subaxial servical spine injury are motor vehicle accidents (50%) and sports-related activities (25%) [8]. Previously reviewed literature and case reports showed very rare pediatric subaxial cervical unilateral facet dislocation (Table 1).

**Table 1:** Summary of published reports on the management of traumatic pediatric unilateral cervical dislocation

Authors & Year	Sex	Patient Age	Level of injured	Injury pattern	Neurological status	Definition of Management	Final Outcome
Faschingbauer M, et al., 2008 [9]	F	12 yrs	C6–7	Traffic accident	No neurological deficit	C6–7 anterior fusion and stabilization, and C5–7 posterior spinous process cerclage wire and fusion	Complete recovery
Parada SA, et al., 2010 [10]	M	9 yrs.	C4–5	Wrestling	No neurological deficit	Manual reduction and stabilization with cervical collar	Complete recovery
Sellin JN, et al., 2014 [6]	F	7 yrs.	C3–4	Fall from height	No neurological deficit	C2–4 posterior cervical fusion, stabilized with lateral mass screws & rod	Complete recovery
Qu W, et al., 2016 [11]	M	5 yrs.	C3–4	Traffic accident	No neurological deficit	C3–4 posterior cervical fusion stabilized	Complete recovery

Knowledge of the anatomical characteristics of pediatric unilateral cervical facet dislocation is very important. Three studies [6, 9, 10] reported three cases of pediatric unilateral cervical facet dislocation without neurological deficit. All reports were performed surgical treatment in school-aged children (5–12 yrs.) and reported a successful clinical outcome. Only one report, Parada et al., 2010 [11] reported a successful clinical outcome by conservative treatment with manual reduction and stabilization with cervical collar.

In our case, the purpose was to highlight the challenges in managing cervical spine injuries in toddlers without neurological deficit. The authors described the management of an acute pediatric unilateral facet dislocation by manual reduction and stabilization with minerva cast. The patient was asymptomatic, had full active cervical motion and good function. The cervical spine showed normal alignment and had healed in radiographs at one-year follow up. Unilateral cervical facet dislocation without neurological deficit in toddlers is very rare. The closed reduction maneuver and applied minerva cast is optional for treatment if successful manual reduction and stabilization with cervical orthosis has a good clinical outcome.

## Data Availability

Data sharing is not applicable to this case report as no datasets were generated or analyzed during the current study.

## Abbreviations

CT: Computed tomography scan
MRI: Magnetic resonance imaging
PICU: Pediatric intensive care unit
SCIWORA: Spinal cord injury without radiographic abnormalities
